# Construction for the useful classes of minimal circular neighbor designs: A comprehensive framework

**DOI:** 10.1016/j.heliyon.2025.e42446

**Published:** 2025-02-04

**Authors:** Khadija Noreen, Javid Shabbir, M.H. Tahir, Akbar Fardos, Rashid Ahmed, Olayan Albalwi

**Affiliations:** a Department of Statistics, The Islamia University of Bahawalpur, Pakistan; b Department of Statistics, University of Wah, Wah Cantt, Pakistan; c Department of Statistics, Faculty of Science, University of Tabuk, Saudi Arabia

**Keywords:** Circular block, Left-neighbor, Partially balanced, Right-neighbor, Weakly balanced

## Abstract

The primary source of bias in the estimation of treatment effect is neighbor effect which can occur in a variety of experiments. This kind of prejudice is reduced by the usage of neighbor balance designs. This article explains the logic behind developing the constructors by using the method of cyclic shifts, which yields sets of shifts for possible combinations of *v* and k to generate minimal circular balanced neighbor designs and their generalized classes when the effects of left-neighbor and right-neighbor are equal. These constructors make it simple to create a number of practical neighbor design classes. The process of creating the constructors will serve as the foundation for the researchers to create additional constructors for novel designs with additional crucial characteristics.

## 1 Introduction

In some experiments, the treatment applied to one trial plot might influence the response on adjoining plots as well as on the plot to which it is applied, such influence is called neighbor effect. In many agricultural experiments, the response from a given plot is affected by treatments applied to neighboring plots (neighbor effect) provided that the plots are adjacent with no gaps. When treatments are varieties, for instance, differences in height or the date of germination may cause neighbor effects, particularly on small plots. Treatments such as fertilizer, water system, or pesticide might spread to adjoining plots causing neighbor effects. In order to avoid the bias in comparing the effects of treatments in such a situation, it is important to ensure that no treatment is unduly advantaged or disadvantaged by its neighbor. A bias is raised in estimating the treatment effects (direct effect) due to neighbor effects. To control neighbor effects, there is a need of designs that will afford protection against the neighbor effects. Designs balanced for neighbor effects provide a tool for local control in biometrics, agriculture, horticulture and forestry. Balanced and strongly balanced neighbor design in minimal circular blocks (MCBND, MCSBND) minimize the bias due to neighbor effects economically. These designs can be obtained only for *v* odd, therefore, generalized classes of (i) MCBNDs such as minimal circular partially balanced and weakly balanced neighbor designs (MCPBNDs-I, MCWBNDs-I), (ii) MCSBNDs such as minimal circular strongly partially balanced and strongly balanced generalized neighbor designs (MCSPBNDs-I, MCSBGNDs-I) are used for *v* even. For better understanding, there are some important definitions. Let *v* treatments are labelled as 0, 1, …, *v*-1. Each treatment of a circular block has one left neighbor and one right neighbor. Considering left-neighbor and right-neighbor effects as same in a circular design:

• If all unordered pairs of treatments appear once (i) excluding pairs of same treatments (0, 0), (1, 1), …, (*v*-1, *v*-1) then it will be MCBND, (ii) including pairs of same treatments then it will be MCSBND, (iii) except the *u* pairs (0, *u*), (1, *u*+1), …, (*u*-1, *v*-1) which do not appear then it will be MCPBND-I, (iv) except the *u* pairs (0, *u*), (1, *u*+1), …, (*u*-1, *v*-1) which appear twice then it will be MCWBND-I, where *u* = *v*/2 with *v* even.
• Considering left-neighbor and right-neighbor effects not same in a circular design if all unordered pair of treatments appear once as left-neighbor and once as right-neighbor (i) excluding pairs of same treatments (0, 0), (1, 1), …, (*v*-1, *v*-1) then it will be MCBND, (iii) including pairs of same treatments except (*v*-1, *v*-1) then it will be MCNSBND.

MCBNDs were initially used in laboratory by Ref. [1] for virus research. [[2], [3], [4], [5]] constructed CBNDs for some different cases through cyclic shifts. [[6], [7], [8], [9]] constructed generalized neighbor designs and their special classes such as GN_2_- and GN_3_- designs. [10,11] used cyclic shifts to compile the catalogues of MCPBNDs for different cases. [12] developed constructors for the complete solution of MCWBNDs-II. [13] developed constructors for the complete solution of MCPBNDs-I then [14] developed constructors to obtain MCPBNDs-II only for *m*(mod 4) ≡ 0 & 1 which were completed by Ref. [15] for the remaining cases of *m*(mod 4) ≡ 2 & 3. [16] developed constructors for the complete solution of MCSPBNDs-I. [17] developed constructors for the complete solution of MCSBGNDs-I & II. [18] developed an R based algorithm which used the constructors for the complete solution of MCBNDs, MCSBNDs and their eight generalized classes. Algorithm was developed on the basis of constructors available in literature to generate the cyclic shifts for MCBNDs and its important generalized classes. The procedure how these constructors were created has not been described in literature. The process of creating the constructors will serve as the foundation for the researchers to create additional constructors for novel designs with additional crucial characteristics. In this article, therefore, method is described how the constructors have been developed which provide the complete solution of MCBNDs, MCSBNDs, MCNSBNDs, MCPBNDs-I, MCSPBNDs-I and MCWBNDs-I in equal and unequal block sizes, assuming left-neighbor and right-neighbor effects as (i) equal, and (ii) unequal for:

• *v* = 2*i*k + C_1_ (equal block size)
• *v* = 2*i*k_1_+ 2γ_1_k_2_+ … + 2γ_h_k_h_ + C_1_ (unequal block sizes), where *i* sets of shifts will be for k_1_ and γ_*j*_ sets for k_*j*_, γ_*j*_ is a whole number and *v* ≥ k_*i*_*.*

## 2 Method of cyclic shifts

Method of cyclic shifts introduced by Ref. [19] is explained here for the construction of MCBNDs, MCSBNDs and their generalized classes.

**Rule I:** Let S_j_ = [$q_{i1}$, $q_{i2}$, …, $q_{i\left(k-1\right)}$] be sets of shifts then (i) S∗ = [$q_{i1}$, $q_{i2}$, …, $q_{i\left(k-1\right)}$, ($q_{i1}$ + $q_{i2}$ + … + $q_{i\left(k-1\right)}$) mod *v*, *v*-q_i1,_ *v*-q_i2,_ …, *v*-q_i(k-1),_ *v*-$[(q_{i1}$ + $q_{i2}$ + … + $q_{i\left(k-1\right)})$ mod *v*] for left and right-neighbor effects same, (ii) S∗ = [$q_{i1}$, $q_{i2}$, …, $q_{i\left(k-1\right)}$, *v*-($q_{i1}$ + $q_{i2}$ + … + $q_{i\left(k-1\right)}$) mod *v*] for left-and right-neighbor effects not same. If 1 ≤ *q*_*ij*_ ≤ *v*-1 and each of 1, 2, …, *v*-1:

• appears once in S∗ then it is MCBND.
• appears once in S∗ except *v*/2 which does not appear then it is MCPBND-I.
• appears once in S∗ except *v*/2 which appears twice then it is MCWBND-I.

If 0 ≤ *q*_*ij*_ ≤ *v*-1, then it will be either MCSBND or one of its generalized classes.

Example 2.1 S = [1,3,4,9] produce MCBNDs for v = 11 and k = 5.

**Proof:** Each of 1, 2, …, 10 appears once in S∗ = [1,3,4,9,6,10,8,7,2,5], so S = [1,3,4,9] produce MCBND.

Take v blocks for S and insert 0, 1, 2, …, *v*-1 in first unit of each block. Add 1(mod v) to each element of first row to complete second row. Similarly, add 3(mod v) to each element of second row to complete third row, and so on in Table 1.

**Table 1:** Blocks generated from S = [1,3,4,9].

Blocks									
B_1_	B_2_	B_3_	B_4_	B_5_	B_6_	B_7_	B_8_	B_9_	B_10_
1	2	3	4	5	6	7	8	9	10
2	3	4	5	6	7	8	9	10	1
5	6	7	8	9	10	1	2	3	4
9	10	1	2	3	4	5	6	7	8
7	8	9	10	1	2	3	4	5	6

### 2.1 Rule II

Let S_a_ = [*q*_a1_, *q*_a2_, …, *q*_a(k-1)_] and S_b_ = [*q*_b1_, *q*_b2_, …, *q*_b(k-2)_]t be the sets of shifts then S∗ = [*q*_a1_, *q*_a2_, …, *q*_a(k-1)_, (*v*-1)-(*q*_a1_+*q*_a2_+ … +*q*_a(k-1)_) mod(*v*-1), *q*_b1_, *q*_b2_, …, *q*_b(k-2)_].

• If 1 ≤ *q*_*ij*_ ≤ *v*-2 and each of 1, 2, …, *v*-2 appears once in S∗ then it is MCBND.
• If 0 ≤ *q*_*ij*_ ≤ *v*-2 and each of 0, 1, …, *v*-2 appears once in S∗ then it is MCNSBND.

Example 2.2 S_1_ = [2,4,5,6] and S_2_ = [1,3]t produce MCBND for v *=* 9, *p*_1_ = 5 and *p*_2_ = 4.

Proof: S∗ = [2,4,5,6,**7**,1,3], Here 1,2,3,4,5,6,7 appears once, hence S_1_ = [2,4,5,6], S_2_ = [1,3]t produce MCBND.

Take *v*-1 blocks for S_1_ = [2,4,5,6] and insert 0, 1, …, *v*-2 in its first row. Add 2 mod (*v*-1) to each element of first row, to get second row. Similarly, add 4, 5 and 6 (mod 8) respectively in Table 2.

**Table 2:** Blocks obtained from S_1_ = [2,4,5,6].

B_1_	B_2_	B_3_	B_4_	B_5_	B_6_	B_7_	B_8_
0	1	2	3	4	5	6	7
2	3	4	5	6	7	0	1
6	7	0	1	2	3	4	5
3	4	5	6	7	0	1	2
1	2	3	4	5	6	7	0

Take more *v*-1 blocks for S_2_ = [1,3]t. Fill it out similarly to how you did in Table 2. In Table 3, add an additional row with *v*-1 as an element of each unit.

**Table 3:** Blocks obtained from S_1_ = [1,3]t.

B_9_	B_10_	B_11_	B_12_	B_13_	B_14_	B_15_	B_16_
0	1	2	3	4	5	6	7
1	2	3	4	5	6	7	0
4	5	6	7	0	1	2	3
8	8	8	8	8	8	8	8

## 3 Logic behind the cyclic shifts method (Rule I) for circular neighbor designs

First we discuss MCBNDs.

### 3.1 MCBNDs and cyclic shifts (Rule I)

Logic behind the cyclic shifts (Rule I) for MCBNDs when left and right-neighbor effects are equal.

• If *v* = 2*m* + 1 then **A** = [1, 2, …, *m*] will produce MCBNDs if sum of **A** is divisible by *v* with *m* = (*v*-1)/2. If sum of **A** is not divisible by *v* then it can be obtained by replacing one or more elements with their respective complements.
• If increase needed in the sum is "*u* = 2*j*+1; *j* (integer) ≥ 0", use "*m* + *j*+1" instead of "*m*-*j*".

Here is an illustration of how to increase the sum of the elements in "**A**" by substituting the value "a" for its complement, "*v*-a," when *v* = 13 and *m* = 6 is given in Table 4.

**Table 4:** Increase in sum of elements by substituting ‘a’ with its complement “*v*-a”.

‘a’	1	2	3 (*m*-3)	4 (*m*-2)	5 (*m*-1)	6 (*m*)
Complement of ‘a’	12	11	10 (*m*+4)	9 (*m*+3)	8 (*m*+2)	7 (*m*+1)
Increase in sum	11	9	7	5	3	1

### 3.2 Logic behind the constructors developed by Ref. [20] for MCBNDs

To make the sum of **A** = [1, 2, …, *m*] divisible by *v*, look at Table 5. Here, **S** stands for the sum of **A**, **R** for the necessary divisibility of **S** by *v*, and **M** = **R**-**S**.

• In *m*(mod 8) ≡ C, C is remainder when *m* is divided by 8. In this study, let *j* = (*m*-C)/8.

**Table 5:** Development of constructors for MCBNDs.

*v*	*m*	A = [1, 2, …, *m*]	S	R	M
9	4	[1,2,3,4]	10	18	8
11	5	[1,2,3,4,5]	15	22	7
13	6	[1,2,3,4,5,6]	21	26	5
15	7	[1,2,3,4,5,6,7]	28	30	2
17	8	[1,2,3,4,5,6,7,8]	36	51	15
19	9	[1,2,3,4,5,6,7,8,9]	45	57	12
21	10	[1,2,3,4,5,6,7,8,9,10]	55	63	8
23	11	[1,2,3,4,5,6,7,8,9,10,11]	66	69	3
25	12	[1,2,3,4,5,6,7,8,9,10,11,12]	78	100	22
27	13	[1,2,3,4,5,6,7,8,9,10,11,12,13]	91	108	17
29	14	[1,2,3,4,5,6,7,8,9,10,11,12,13,14]	105	116	11
31	15	[1,2,3,4,5,6,7,8,9,10,11,12,13,14,15]	120	124	4
33	16	[1,2,3,4,5,6,7,8,9,10,11,12,13,14,15,16]	136	165	29
35	17	[1,2,3,4,5,6,7,8,9,10,11,12,13,14,15,16,17]	153	175	22
37	18	[1,2,3,4,5,6,7,8,9,10,11,12,13,14,15,16,17,18]	171	185	14
39	19	[1,2,3,4,5,6,7,8,9,10,11,12,13,14,15,16,17,18,19]	190	195	5
41	20	[1,2,3,4,5,6,7,8,9,10,11,12,13,14,15,16,17,18,19,20]	210	246	36
43	21	[1,2,3,4,5,6,7,8,9,10,11,12,13,14,15,16,17,18,19,20, 21]	231	258	27
45	22	[1,2,3,4,5,6,7,8,9,10,11,12,13,14,15,16,17,18,19,20,21,22]	253	270	17
47	23	[1,2,3,4,5,6,7,8,9,10,11,12,13,14,15,16,17,18,19,20,21,22,23]	276	282	6
49	24	[1,2,3,4,5,6,7,8,9,10,11,12,13,14,15,16,17,18,19,20,21,22,23,24]	300	343	43
51	25	[1,2,3,4,5,6,7,8,9,10,11,12,13,14,15,16,17,18,19,20,21,22,23,24, 25]	325	357	32
53	26	[1,2,3,4,5,6,7,8,9,10,11,12,13,14,15,16,17,18,19,20,21,22,23,24, 25,26]	351	371	20
55	27	[1,2,3,4,5,6,7,8,9,10,11,12,13,14,15,16,17,18,19,20,21,22,23,24, 25,26,27]	378	385	7
57	28	[1,2,3,4,5,6,7,8,9,10,11,12,13,14,15,16,17,18,19,20,21,22,23,24, 25,26,27,28]	406	456	50
59	29	[1,2,3,4,5,6,7,8,9,10,11,12,13,14,15,16,17,18,19,20,21,22,23,24, 25,26,27,28,29]	435	472	37
61	30	[1,2,3,4,5,6,7,8,9,10,11,12,13,14,15,16,17,18,19,20,21,22,23,24, 25,26,27,28,29,30]	465	488	23
63	31	[1,2,3,4,5,6,7,8,9,10,11,12,13,14,15,16,17,18,19,20,21,22,23,24, 25,26,27,28,29,30,31]	496	504	8
65	32	[1,2,3,4,5,6,7,8,9,10,11,12,13,14,15,16,17,18,19,20,21,22,23,24, 25,26,27,28,29,30,31,32]	528	585	57
67	33	[1,2,3,4,5,6,7,8,9,10,11,12,13,14,15,16,17,18,19,20,21,22,23,24, 25,26,27,28,29,30,31,32,33]	561	603	42
69	34	[1,2,3,4,5,6,7,8,9,10,11,12,13,14,15,16,17,18,19,20,21,22,23,24, 25,26,27,28,29,30,31,32,33,34]	595	621	26
71	35	[1,2,3,4,5,6,7,8,9,10,11,12,13,14,15,16,17,18,19,20,21,22,23,24, 25,26,27,28,29,30,31,32,33,34,35]	630	639	9
73	36	[1,2,3,4,5,6,7,8,9,10,11,12,13,14,15,16,17,18,19,20,21,22,23,24, 25,26,27,28,29,30,31,32,33,34,35,36]	666	730	64
75	37	[1,2,3,4,5,6,7,8,9,10,11,12,13,14,15,16,17,18,19,20,21,22,23,24, 25,26,27,28,29,30,31,32,33,34,35,36,37]	703	750	47

**Table 6:** *m*(mod 8) ≡ 0 and *j* = *m*/8.

*v*	*m*	*j*	A = [1, 2, …, *m*]	Sum	Required	Add	Pattern
17	8	1	[**1**,2,3,4,5,6,7,8]	36	51	15	15
33	16	2	[1,**2**,3,4,5,6,7,8,9,10,11,12,13,14,15,16]	136	165	29	29
49	24	3	[1,2,**3**,4,5,6,7,8,9,10,11,12,13,14,15,16,17, 18,19,20,21,22,23,24]	300	343	43	43
65	32	4	[1,2,3,**4**,5,6,7,8,9,10,11,12,13,14,15,16,17, 18,19,20,21,22,23,24,25,26,27,28,29,30,31,32]	528	585	57	57

**Table 7:** *m*(mod 8) ≡ 1 and *j* = (*m*-1)/8.

*v*	*m*	*j*	A = [1, 2, …, *m*]	Sum	Required	Add	Pattern
19	9	1	[1,2,3,**4**,5,6,7,8,**9**]	45	57	12	11 + 1
35	17	2	[1,2,3,4,5,6,**7**,8,9,10,11,12,13,14,15,16,**17**]	153	175	22	21 + 1
51	25	3	[1,2,3,4,5,6,7,8,9,**10**,11,12,13,14,15,16,17, 18,19,20,21,22,23,24,**25**]	325	357	32	31 + 1
67	33	4	[1,2,3,4,5,6,7,8,9,10,11,12,**13**,14,15,16,17, 18,19,20,21,22,23,24,25,26,27,28,29,30,31,32,**33**]	561	603	42	41 + 1

**Table 8:** *m*(mod 8) ≡ 2 and *j* = (*m*-2)/8.

*v*	*m*	*j*	A = [1, 2, …, *m*]	Sum	Required	Add	Pattern
21	10	1	[1,2,3,4,5,6,**7**,8,9,**10**]	55	63	8	7 + 1
37	18	2	[1,2,3,4,5,6,7,8,9,10,11,**12**,13,14,15,16,17, **18**]	171	185	14	13 + 1
53	26	3	[1,2,3,4,5,6,7,8,9,10,11,12,13,14,15,16,**17**, 18,19,20,21,22,23,24,25,**26**]	351	371	20	19 + 1
69	34	4	[1,2,3,4,5,6,7,8,9,10,11,12,13,14,15,16,17, 18,19,20,21,**22**,23,24,25,26,27,28,29,30,31,32,33,**34**]	595	621	26	25 + 1

**Table 9:** *m*(mod 8) ≡ 3 and *j* = (*m*-3)/8.

*v*	*m*	*j*	A = [1, 2, …, *m*]	Sum	Required	Add	Pattern
7	3	0	[1,2,**3**]	6	7	1	1
23	11	1	[1,2,3,4,5,6,7,8,9,**10**,11]	66	69	3	3
39	19	2	[1,2,3,4,5,6,7,8,9,10,11,12,13,14,15,16,**17**, 18,19]	190	195	5	5
55	27	3	[0,1,2,3,4,5,6,7,8,9,10,11,12,13,14,15,16,17,18,19,20,21,22,23,**24**,25,26,27]	378	385	7	7
71	35	4	[0,1,2,3,4,5,6,7,8,9,10,11,12,13,14,15,16,17,18,19,20,21,22,23,24,25,26,27,28,29,30,**31**,32,33,34,35]	630	639	9	9

**Table 10:** *m*(mod 8) ≡ 4 and *j* = (*m*-4)/8.

*v*	*m*	*j*	A = [1, 2, …, *m*]	Sum	Required	Add	Pattern
9	4	0	[**1**,2,3,**4**]	10	18	8	7 + 1
25	12	1	[1,**2**,3,4,5,6,7,8,9,10,11,**12**]	78	100	22	21 + 1
41	20	2	[1,2,**3**,4,5,6,7,8,9,10,11,12,13,14,15,16,17, 18,19,**20**]	210	246	36	35 + 1
57	28	3	[1,2,3,**4**,5,6,7,8,9,10,11,12,13,14,15,16,17, 18,19,20,21,22,23,24,25,26,27,**28**]	406	456	50	49 + 1
73	36	4	[1,2,3,4,**5**,6,7,8,9,10,11,12,13,14,15,16,17, 18,19,20,21,22,23,24,25,26,27,28,29,30,31,32,33,34,35,**36**]	666	730	64	63 + 1

**Table 11:** *m*(mod 8) ≡ 5 and *j* = (*m*-5)/8.

*v*	*m*	*j*	A = [1, 2, …, *m*]	Sum	Required	Add	Pattern
11	5	0	[1,**2**,3,4,5]	15	22	7	7
27	13	1	[1,2,3,4,**5**,6,7,8,9,10,11,12,13]	91	108	17	17
43	21	2	[1,2,3,4,5,6,7,**8**,9,10,11,12,13,14,15,16,17, 18,19,20,21]	231	258	27	27
59	29	3	[1,2,3,4,5,6,7,8,9,10,**11**,12,13,14,15,16,17, 18,19,20,21,22,23,24,25,26,27,28,29]	435	472	37	37
75	37	4	[1,2,3,4,5,6,7,8,9,10,11,12,13,**14**,15,16,17, 18,19,20,21,22,23,24,25,26,27,28,29,30,31,32,33,34,35,36,37]	703	750	47	47

**Table 12:** *m*(mod 8) ≡ 6 and *j* = (*m*-6)/8.

*v*	*m*	*j*	A = [1, 2, …, *m*]	Sum	Required	Add	Pattern
13	6	0	[1,2,3,**4**,5,6]	21	26	5	5
29	14	1	[1,2,3,4,5,6,7,8,**9**,10,11,12,13,14]	105	116	11	11
45	22	2	[1,2,3,4,5,6,7,8,9,10,11,12,13,**14**,15,16,17, 18,19,20,21,22]	253	270	17	17
61	30	3	[1,2,3,4,5,6,7,8,9,10,11,12,13,14,15,16,17, 18,**19**,20,21,22,23,24,25,26,27,28,29,30]	465	488	23	23

**Table 13:** *m*(mod 8) ≡ 7 and *j* = (*m*-7)/8.

*v*	*m*	*j*	A = [1, 2, …, *m*]	Sum	Required	Add	Pattern
15	7	0	[1,2,3,4,5,6,**7**] **Cannot be obtained**	28	30	2	1 + 1
31	15	1	[1,2,3,4,5,6,7,8,9,10,11,12,13,**14**,**15**]	120	124	4	3 + 1
47	23	2	[1,2,3,4,5,6,7,8,9,10,11,12,13,14,15, 16,17,18,19,20,**21**,22,**23**]	276	282	6	5 + 1
63	31	3	[1,2,3,4,5,6,7,8,9,10,11,12,13,14,15, 16,17,18,19,20,21,22,23,24,25,26, 27,**28**,29,30,**31**]	496	504	8	7 + 1

#### 3.2.1 Table 6, Table 7, Table 8, Table 9, Table 10, Table 11, Table 12, Table 13 show how the values of *m* in Table 5 are arranged, showing a common pattern in R

• Hence, replacing *j* with 2*m*-*j*+1 in **A**, resultant **A**_**1**_ = [1, 2, …, (*j*-1), (*j*+1), (*j*+2), …, *m*, (2*m*+1-*j*)] is the constructor to produce MCBNDs when *m*(mod 8) ≡ 0, *j* = *m*/8 with *j* *≥* 1.
• Replacing *m* & and 3*j*+1 with (*m*+1) & 2*m*-3*j* in **A**, resultant **A**_**2**_ = [1, 2, …, 3*j*, (3*j*+2), (3*j*+3), …, *m*-1, *m*+1, (2*m*-3*j*)] is the constructor to produce MCBNDs when *m*(mod 8) ≡ 1, *j* = (*m*-1)/8 with *j* *≥* 1.
• Replacing *m* & 5*j*+2 with (*m*+1) & 2*m*-5*j*-1 in **A**, resultant **A**_**3**_ = [1, 2, …, (5*j*+1), (5*j*+3), (5*j*+4), …, *m*-1, *m*+1, (2*m*-5*j*-1)] is the constructor to produce MCBNDs when *m*(mod 8) ≡ 2, *j* = (*m*-2)/8 with *j* *≥* 1.
• Replacing (*m*-*j*) with *m*+(*j*+1) in **A**, resultant **A**_**4**_ = [1, 2, …, (*m*-*j*-1), (*m*-*j*+1), (*m*-*j*+2), …, *m*, (*m* + *j*+1)] is the constructor to generate MCBNDs when *m*(mod 8) ≡ 3 and *j* = (*m*-3)/8 with *j* *≥* 0.
• Replacing *m* & *j*+1 with (*m*+1) & 2*m*-*j* in **A**, resultant **A**_**5**_ = [1, 2, …, *j*, (*j*+2), (*j*+3), …, *m*-1, *m*+1, (2*m*-*j*)] is the constructor to generate MCBNDs when *m*(mod 8) ≡ 4, *j* = (*m*-4)/8 with *j* *≥* 0.
• Replacing 3*j*+2 with 2*m*-3*j*-1 in **A**, resultant **A**_**6**_ = [1, 2, …, (3*j*+1), (3*j*+3), (3*j*+4), …, *m*, (2*m*-3*j*-1)] is the constructor to generate MCBNDs when *m*(mod 8) ≡ 5, *j* = (*m*-5)/8 with *j* *≥* 0.
• Replacing 5*j*+4 with 2*m*-5*j*-3 in **A**, resultant **A**_**7**_ = [1, 2, …, (5*j*+3), (5*j*+5), (5*j*+6), …, *m*, (2*m*-5*j*-3)] is the constructor to generate MCBNDs when *m*(mod 8) ≡ 6, *j* = (*m*-6)/8 with *j* *≥* 0.
• Replacing *m* & (*m*-*j*) with (*m*+1) **&** *m*+(*j*+1) in **A**, resultant **A**_**8**_ = [1, 2, …, (*m*-*j*-1), (*m*-*j*+1), (*m*-*j*+2), …, *m*-1, *m*+1, (*m* + *j*+1)] is the constructor to generate MCBNDs when *j* = (*m*-7)/8, *m*(mod 8) ≡ 7 with *j* *≥* 1.
• Adding "0" to the constructors of MCBNDs yields constructors for MCSBNDs.

### 3.3 MCPBNDs-I and cyclic shifts (Rule I)

Logic behind the Rule I for MCPBNDs-I when left and right-neighbor effects are equal.

• If *v* = 2*m* + 2 then **B** = [1, 2, …, *m*] will produce MCPBNDs-I if sum of **B** is divisible by *v* with *m* = (*v*-2)/2. If sum of **B** is not divisible by *v* then it can be obtained by replacing one or more elements with their respective complements.
• If increase needed in the sum is "*u* = 2*j*; *j* (integer) ≥ 1", use "*m*+(*j*+2)" instead of "*m*-*j*". Increase of "*u* = 2*j*+1; *j* (integer) ≥ 0" in the sum is not possible.

Here is an illustration of how to increase the sum of the elements in "**B**" by substituting the value "a" for its complement, "*v*-a," when *v* = 14 and *m* = 6.

**Table:** 

‘a’	1	2	3 (*m*-3)	4 (*m*-2)	5 (*m*-1)	6 (*m*)
**Complement of ‘a’**	13	12	11 (*m*+5)	10 (*m*+4)	9 (*m*+3)	8 (*m*+2)
**Increase in Sum**	12	10	8	6	4	2

### 3.4 Logic behind the constructors developed by Nadeem et al. [13] for MCPBNDs-I

To make the sum of **B** = [1, 2, …, *m*] divisible by *v*, consider Table 14.

**Table 14:** Development of constructors for MCPBNDs-I.

*v*	*m*	A = [1, 2, …, *m*]	S	R	M
8	3	[1,2,3]	6	8	2
10	4	[1,2,3,4]	10	Divisible	–
12	5	[1,2,3,4,5]	15	24	9
14	6	[1,2,3,4,5,6]	21	28	7
16	7	[1,2,3,4,5,6,7]	28	32	4
18	8	[1,2,3,4,5,6,7,8]	36	Divisible	–
20	9	[1,2,3,4,5,6,7,8,9]	45	60	15
22	10	[1,2,3,4,5,6,7,8,9,10]	55	66	11
24	11	[1,2,3,4,5,6,7,8,9,10,11]	66	72	6
26	12	[1,2,3,4,5,6,7,8,9,10,11,12]	78	Divisible	–
28	13	[1,2,3,4,5,6,7,8,9,10,11,12,13]	91	112	21
30	14	[1,2,3,4,5,6,7,8,9,10,11,12,13,14]	105	120	15
32	15	[1,2,3,4,5,6,7,8,9,10,11,12,13,14,15]	120	128	8
34	16	[1,2,3,4,5,6,7,8,9,10,11,12,13,14,15,16]	136	Divisible	–
36	17	[1,2,3,4,5,6,7,8,9,10,11,12,13,14,15,16,17]	153	180	27
38	18	[1,2,3,4,5,6,7,8,9,10,11,12,13,14,15,16,17,18]	171	190	19
40	19	[1,2,3,4,5,6,7,8,9,10,11,12,13,14,15,16,17,18,19]	190	200	10
42	20	[1,2,3,4,5,6,7,8,9,10,11,12,13,14,15,16,17,18,19,20]	210	Divisible	–
44	21	[1,2,3,4,5,6,7,8,9,10,11,12,13,14,15,16,17,18,19,20,21]	231	264	33
46	22	[1,2,3,4,5,6,7,8,9,10,11,12,13,14,15,16,17,18,19,20,21,22]	253	276	23
48	23	[1,2,3,4,5,6,7,8,9,10,11,12,13,14,15,16,17,18,19,20,21,22,23]	276	288	12
50	24	[1,2,3,4,5,6,7,8,9,10,11,12,13,14,15,16,17,18,19,20,21,22, 23,24]	300	Divisible	–

**Table 15:** *m*(mod 4) ≡ 0.

*v*	*m*	B = [1, 2, …, *m*]	S	R
10	4	[1,2,3,4]	10	Divisible
18	8	[1,2,3,4,5,6,7,8]	36	Divisible
26	12	[1,2,3,4,5,6,7,8,9,10,11,12]	78	Divisible
34	16	[1,2,3,4,5,6,7,8,9,10,11,12,13,14,15,16]	136	Divisible
42	20	[1,2,3,4,5,6,7,8,9,10,11,12,13,14,15,16,17,18,19,20]	210	Divisible
50	24	[1,2,3,4,5,6,7,8,9,10,11,12,13,14,15,16,17,18,19,20,21,22,23,24]	300	Divisible

**Table 16:** *m*(mod 4) ≡ 3 and *i* = (*m*+1)/4.

*v*	*m*	*i*	B = [1, 2, …, *m*]	S	R	M
8	3	1	[1,2,**3**]	6	8	2
16	7	2	[1,2,3,4,5,**6**,7]	28	32	4
24	11	3	[1,2,3,4,5,6,7,8,**9**,10,11]	66	72	6
32	15	4	[1,2,3,4,5,6,7,8,9,10,11,**12**,13,14,15]	120	128	8
40	19	5	[1,2,3,4,5,6,7,8,9,10,11,12,13,14,**15**,16,17,18,19]	190	200	10
48	23	6	[1,2,3,4,5,6,7,8,9,10,11,12,13,14,15,16,17,**18**,19,20,21, 22,23]	276	288	12

**Table 17:** *m*(mod 4) ≡ 1 & 2.

*v*	*m*	B = [1, 2, …, *m*]	S	R	M
12	5	[1,2,3,4,5]	15	24	9
14	6	[1,2,3,4,5,6]	21	28	7
20	9	[1,2,3,4,5,6,7,8,9]	45	60	15
22	10	[1,2,3,4,5,6,7,8,9,10]	55	66	11
28	13	[1,2,3,4,5,6,7,8,9,10,11,12,13]	91	112	21
30	14	[1,2,3,4,5,6,7,8,9,10,11,12,13,14]	105	120	15
36	17	[1,2,3,4,5,6,7,8,9,10,11,12,13,14,15,16,17]	153	180	27
38	18	[1,2,3,4,5,6,7,8,9,10,11,12,13,14,15,16,17,18]	171	190	19
44	21	[1,2,3,4,5,6,7,8,9,10,11,12,13,14,15,16,17,18,19,20,21]	231	264	33
46	22	[1,2,3,4,5,6,7,8,9,10,11,12,13,14,15,16,17,18,19,20,21,22]	253	276	23

#### 3.4.1 Table 15, Table 16, Table 17 show how the values of *m* in Table 14 are arranged, showing a common pattern in R

• Hence, the constructor to generate MCPBNDs-I for *m* (mod 4) ≡ 0 is **B** = [1, 2, …, *m*].
• The constructor will generate MCPBNDs-I for *m*(mod 4) ≡ 3 if the value "3*i*" in C = [1, 2, …, *m*] is substituted with "*v*-3*i.*"
• Adding "0" to the constructors of MCPBNDs-I yields constructors for MCSPBNDs-I.
• Since M is odd for *m*(mod 4) ≡ 1 & 2, the sum of **B** = [1, 2, …, *m*] cannot be made divisible by *v* by substituting the complements of values(s). Consequently, for *m*(mod 4) ≡ 1 & 2, MCPBNDs-I cannot be obtained.

### 3.5 MCWBNDs-I and cyclic shifts (Rule I)

Logic behind the Rule I for MCWBNDs-I when left and right-neighbor effects are equal.

• If *v* = 2*m* + 2 then **C** = [1, 2, …, *m*+1] will produce MCWBNDs-I if sum of **C** is divisible by *v* with *m* = (*v*-2)/2. If sum of **C** is not divisible by *v* then it can be obtained by replacing some elements with their respective complements.

### 3.6 Logic behind the constructors developed by Rasheed et al. [21] for MCWBNDs-I

To make the sum of **C** = [1, 2, …, *m*+1] divisible by *v*, consider Table 18.

**Table 18:** Development of constructors for MCWBNDs-I.

*v*	*m*	C = [1, 2, …, *m*+1]	S	R	M
6	2	[1,2,3]	6	Divisible	–
8	3	[1,2,3,4]	10	16	6
10	4	[1,2,3,4,5]	15	20	5
12	5	[1,2,3,4,5,6]	21	24	3
14	6	[1,2,3,4,5,6,7]	28	Divisible	–
16	7	[1,2,3,4,5,6,7,8]	36	48	12
18	8	[1,2,3,4,5,6,7,8,9]	45	54	9
20	9	[1,2,3,4,5,6,7,8,9,10]	55	60	5
22	10	[1,2,3,4,5,6,7,8,9,10,11]	66	Divisible	–
24	11	[1,2,3,4,5,6,7,8,9,10,11,12]	78	96	18
26	12	[1,2,3,4,5,6,7,8,9,10,11,12,13]	91	104	13
28	13	[1,2,3,4,5,6,7,8,9,10,11,12,13,14]	105	112	7
30	14	[1,2,3,4,5,6,7,8,9,10,11,12,13,14,15]	120	Divisible	–
32	15	[1,2,3,4,5,6,7,8,9,10,11,12,13,14,15,16]	136	160	24
34	16	[1,2,3,4,5,6,7,8,9,10,11,12,13,14,15,16,17]	153	170	17
36	17	[1,2,3,4,5,6,7,8,9,10,11,12,13,14,15,16,17,18]	171	180	9
38	18	[1,2,3,4,5,6,7,8,9,10,11,12,13,14,15,16,17,18,19]	190	Divisible	–
40	19	[1,2,3,4,5,6,7,8,9,10,11,12,13,14,15,16,17,18,19,20]	210	240	30
42	20	[1,2,3,4,5,6,7,8,9,10,11,12,13,14,15,16,17,18,19,20, 21]	231	252	21
44	21	[1,2,3,4,5,6,7,8,9,10,11,12,13,14,15,16,17,18,19,20, 21,22]	253	264	11
46	22	[1,2,3,4,5,6,7,8,9,10,11,12,13,14,15,16,17,18,19,20,21, 22,23]	276	Divisible	–
48	23	[1,2,3,4,5,6,7,8,9,10,11,12,13,14,15,16,17,18,19,20,21, 22,23,24]	300	336	36
50	24	[1,2,3,4,5,6,7,8,9,10,11,12,13,14,15,16,17,18,19,20,21, 22,23,24,25]	325	350	25

**Table 19:** *m*(mod 4) ≡ 2.

*v*	*m*	C = [1, 2, …, *m*+1]	S	R
6	2	[1,2,3]	6	Divisible
14	6	[1,2,3,4,5,6,7]	28	Divisible
22	10	[1,2,3,4,5,6,7,8,9,10,11]	66	Divisible
30	14	[1,2,3,4,5,6,7,8,9,10,11,12,13,14,15]	120	Divisible
38	18	[1,2,3,4,5,6,7,8,9,10,11,12,13,14,15,16,17,18,19]	190	Divisible
46	22	[1,2,3,4,5,6,7,8,9,10,11,12,13,14,15,16,17,18,19,20,21,22,23]	276	Divisible

**Table 20:** *m*(mod 4) ≡ 3 and *i* = (*m*+1)/4.

*v*	*m*	*i*	D = [1, 2, …, *m*, *m*+1]	S	R	D
8	3	1	[**1**,2,3,4]	10	16	6
16	7	2	[1,**2**,3,4,5,6,7,8]	28	32	12
24	11	3	[1,2,**3**,4,5,6,7,8,9,10,11,12]	66	72	18
32	15	4	[1,2,3,**4**,5,6,7,8,9,10,11,12,13,14,15,16]	120	128	24
40	19	5	[1,2,3,4,**5**,6,7,8,9,10,11,12,13,14,15,16,17,18,19,20]	190	200	30
48	23	6	[1,2,3,4,5,**6**,7,8,9,10,11,12,13,14,15,16,17,18,19,20,21,22,23,24]	276	288	36

**Table 21:** *m*(mod 4) ≡ 0 and 1.

*v*	*m*	D = [1, 2, …, *m*+1]	S	R	M
10	4	[1,2,3,4,5]	15	20	5
12	5	[1,2,3,4,5,6]	21	24	3
18	8	[1,2,3,4,5,6,7,8,9]	45	54	9
20	9	[1,2,3,4,5,6,7,8,9,10]	55	60	5
26	12	[1,2,3,4,5,6,7,8,9,11,12,13]	91	104	13
28	13	[1,2,3,4,5,6,7,8,9,10,11,12,13,14]	105	112	7
34	16	[1,2,3,4,5,6,7,8,9,10,11,12,13,14,15,16,17]	153	170	17
36	17	[1,2,3,4,5,6,7,8,9,10,11,12,13,14,15,16,17,18]	171	180	9
42	20	[1,2,3,4,5,6,7,8,9,10,11,12,13,14,15,16,17,18,19,20,21]	231	252	21
44	21	[1,2,3,4,5,6,7,8,9,10,11,12,13,14,15,16,17,18,19,20,21,22]	253	264	11
50	24	[1,2,3,4,5,6,7,8,9,10,11,12,13,14,15,16,17,18,19,20,21,22,23,24, 25]	325	350	25

#### 3.6.1 Table 19, Table 20, Table 21 show how the values of *m* in Table 18 are arranged, showing a common pattern in R

• Hence, the constructor to generate MCWBNDs-I for *m*(mod 4) ≡ 2 is **C** = [1, 2, …, *m*+1].
  • The constructor will generate MCWBNDs-I for *m*(mod 4) ≡ 3 if the value "*i*" in C = [1, 2, …, *m*+1] is substituted with "*v*-*i.*"
• Since M is odd for *m*(mod 4) ≡ 0 & 1, the sum of **C** = [1, 2, …, *m*+1] cannot be made divisible by *v* by substituting the complements of values(s). Consequently, for *m*(mod 4) ≡ 0 & 1, MCWBNDs-I cannot be obtained.

### 3.7 Developing sets of shifts from the constructors derived from Rule I

• Divide the selected constructor into *i* groups of k values for *v* = 2*i*k + C_1_ (equal block size), with sum of each group being divisible by *v.*
• For *v* = 2*i*k_1_+ 2γ_1_k_2_+ … + 2γ_h-1_ k_h_ + C_1_ (unequal block sizes), divide the selected constructor into *i* groups of k_1_, γ_1_ groups of k_2_, γ_2_ groups of k_3_, …, γ_h-1_ groups of k_h_ values with sum of each group being divisible by *v.*
• Removing one value (any value) from every group, resulting will produce the sets of shift, see Rasheed et al. [21].

Example 3.1 Sets of shifts for MCBND for v = 51 and k = 5 can be obtained from the constructor **A** = [1,2, …,8,9,**41**,11,12, …,24,**26**] as: Consider v = 2*i*k+1, here i = 5. Divide **A** into five groups each of size 5 with their sum divisible by 51.

G_1_ = (3,4,5,13,26), G_2_ = (6,7,8,14,16), G_3_ = (2,9,11,12,17), G_4_ = (18,19,20,21,24),
G_5_ = (1,15,22,23,41)

Deleting one value (any) from each group, following are the sets of shifts which provide the MCBND for v = 51 and k = 5.

S_1_ = [3,4,13,26], S_2_ = [6,8,14,16], S_3_ = [2,9,12,17], S_4_ = [18,20,21,24],
S_5_ = [1,15,23,41]

Similarly sets of shifts to obtain MCBND can be developed from this constructor for the following cases with 3 ≤ k_h_ ≤ 10 in blocks of:

(a) Two different sizes, considering v = 2*i*k_1_+2γ_1_k_2_+1.
  • k_1_ = 7, k_2_ = 4, here i = 3, γ_1_ = 1.
    S_1_ = [4,7,14,21,24,26], S_2_ = [3,9,11,12,18,41], S_3_ = [2,15,16,17,19,20],
    S_4_ = [1,5,23] are the sets of shifts to obtain MCBND for v = 51, k_1_ = 7, k_2_ = 4.
  • k_1_ = 7, k_2_ = 6, here i = 1, γ_1_ = 3.
  • k_1_ = 9, k_2_ = 7, here i = 2, γ_1_ = 1.
  • k_1_ = 9, k_2_ = 8, here i = 1, γ_1_ = 2.
  • k_1_ = 10, k_2_ = 5, here i = 2, γ_1_ = 1.
  • k_1_ = 10, k_2_ = 5, here i = 1, γ_1_ = 3.
(b) Three different sizes, considering v = 2*i*k_1_+2γ_1_k_2_+2γ_2_k_3_+1.
  • k_1_ = 6, k_2_ = 4, k_3_ = 3, here i = 3, γ_1_ = 1, γ_2_ = 1.
    S_1_ = [2,3,6,9,26], S_2_ = [1,11,15,22,41], S_3_ = [13,14,18,20,21], S_4_ = [7,17,19],
    S_5_ = [4,24] are the sets of shifts to obtain MCBND for v = 51, k_1_ = 6, k_2_ = 4, k_3_ = 3.
  • k_1_ = 8, k_2_ = 7, k_3_ = 5, here i = 1, γ_1_ = 1, γ_2_ = 2.
  • k_1_ = 9, k_2_ = 6, k_3_ = 5, here i = 1, γ_1_ = 1, γ_2_ = 2.
  • k_1_ = 9, k_2_ = 8, k_3_ = 4, here i = 1, γ_1_ = 1, γ_2_ = 2.

### 3.8 Rule I-based constructors for MCBNDs and MCSBNDs in which the effects of left and right neighbors are not equal

On the basis of Rule I, constructors [1, 2, …, *v*-1] and [0, 1, …, *v*-1] produce the full solutions of MCBNDs and MCSBNDs, respectively for the following cases of *v* odd, given that left and right-neighbor effects are not equal.

• *v* = *i*k + C_1_ (equal block size)
• *v* = *i*k_1_+ γ_1_k_2_+ … + γ_h-1_ k_h_ + C_1_ (unequal block sizes), where *i* sets of shifts will be for k_1_ and γ_*j*_ sets for k_*j*_, where γ_*j*_ are the whole numbers and C_1_ is 1 for MCBNDs and −1 for MCSBNDs.

The constructors (as presented by Ref. [22]: [1, 2, …, (*v*-2)/2, (*v*+2)/2, (*v*+2)/2, …, *v*-1], [0, 1, …, (*v*-2)/2, (*v*+2)/2, (*v*+2)/2, …, *v*-1], [1, 2, …, *v*-1, *v*/2] and [0, 1, 2, …, *v*-1, *v*/2] provide the full solution of MCPBNDs-I, MCSPBNDs-I, MCWBNDs-I, and MCSBGNDs-I, respectively, based on Rule I for *v* even with:

• *v* = *i*k + D_1_ (equal block size)
• *v* = *i*k_1_+ γ_1_k_2_+ … + γ_h-1_ k_h_ + D_1_ (unequal block sizes), where *i* sets of shifts will be for k_1_ and γ_*j*_ sets for k_*j*_, where D_1_ = 2, 1, 0 & −1 for MCPBNDs-I, MCSPBNDs-I, MCWBNDs-I and F_1_ = −1 for MCSBGNDs-I respectively.

### 3.9 Rule II-based constructors for MCBNDs and MCNSBNDs in which the effects of left and right neighbors are not equal

On the basis of Rule II, constructors [1, 2, …, *v*-2] and [0, 1, …, *v*-2] produce the full solutions of MCBNDs and MCNSBNDs, respectively for the following cases, given that left and right-neighbor effects are not equal.

• *v* *=* *i*k + D_1_ (equal block size)
• *v* = *i*k_1_+ γ_1_k_2_+ … + γ_h-1_ k_h_ + D_1_ (unequal block sizes), where *i* sets of shifts will be for k_1_ and γ_*j*_ sets for k_*j*_, where γ_*j*_ are the whole numbers, D_1_ = 0 for MCBNDs and D_1_ = −1 for MCNSBNDs.

### 3.10 Developing sets of shifts from constructors derived from Rule II

• Split the chosen constructor into (*i*-1) groups of k values for *v* = *i*k + D_1_ (equal block size). The sum of each group should be divisible by (*v*-1); the remaining k-2 values will be in the *i*th group.
• For *v* = *i*k_1_+ γ_1_k_2_+ … + γ_h-1_ k_h_ + D_1_ (unequal block sizes), split the chosen constructor into *i* groups of k_1_, γ_1_ groups of k_2_, γ_2_ groups of k_3_, …, γ_h_ −2 groups of k_h_-1 values, with the sum of each group being divisible by (*v*-1). The remaining k_h_-2 values will be in the hth group.
• Removing one value (any value) from every group, with the exception of the final group that contains k_h_ −2 values, will produce the sets of shift, see Hassan et al. [23].

Example 3.2 The constructor B = [1, 2, …, 18] yields sets of shifts for MCBND (when left and right-neighbor effects are not the same) for v = 20 and k = 5 as follows:

Consider v = *i*k, here i = 4. Divide the selected constructor into three groups of 5 values with sum of each group divisible by 19, 4th group will contain the remaining three values.

G_1_ = (1,2,3,6,7), G_2_ = (4,8,17,10,18), G_3_ = (5,16,11,12,13), G_4_ = (9,14,15)

For v = 20 and k = 5, we obtain the following sets of shifts by deleting the smallest value from the first three groups while leaving the fourth unchanged.

S_1_ = [2,3,6,7], S_2_ = [8,10,17,18], S_3_ = [11,12,13,16], S_4_ = [9,14,15]t

This constructor can also be used to create sets of shifts to obtain MCBND for the following cases where 3 ≤ k_h_ ≤ 10 in blocks of:

(1) Equal sizes, considering v = *i*k.
  • k = 4, here i = 5.
  • k = 10, here i = 2.
(2) Two different sizes, considering v = *i*k_1_+γ_1_k_2_.
  • k_1_ = 6, k_2_ = 4, here i = 2 and γ_1_ = 2.
  • k_1_ = 7, k_2_ = 3, here i = 2 and γ_1_ = 2.
  • k_1_ = 7, k_2_ = 6, here i = 2 and γ_1_ = 1.
  • k_1_ = 8, k_2_ = 4, here i = 2 and γ_1_ = 1.
  • k_1_ = 8, k_2_ = 4, here i = 1 and γ_1_ = 3.
  • k_1_ = 8, k_2_ = 6, here i = 1 and γ_1_ = 2.
  • k_1_ = 10, k_2_ = 5, here i = 1 and γ_1_ = 2.
    S_1_ = [1,2,3,4,5,6,7,16,18], S_2_ = [8,9,12,13,10], S_3_ = [8,15,17]t are the sets of shifts to obtain MCBND for v = 20, k_1_ = 10, k_2_ = 5.
(3) Three different sizes, considering v = *i*k_1_+γ_1_k_2_+γ_2_k_3_.
  • k_1_ = 6, k_2_ = 4, k_3_ = 3, here i = 2, γ_1_ = 2 & γ_2_ = 2.
  • k_1_ = 7, k_2_ = 5, k_3_ = 4, here i = 1, γ_1_ = 1 & γ_2_ = 2.
  • k_1_ = 8, k_2_ = 6, k_3_ = 3, here i = 1, γ_1_ = 1 & γ_2_ = 2.
  • k_1_ = 8, k_2_ = 7, k_3_ = 5, here i = 1, γ_1_ = 1 & γ_2_ = 1.
  • k_1_ = 9, k_2_ = 5, k_3_ = 3, here i = 1, γ_1_ = 1 & γ_2_ = 2.
  • k_1_ = 9, k_2_ = 8, k_3_ = 3, here i = 1, γ_1_ = 1 & γ_2_ = 1.
  • k_1_ = 9, k_2_ = 7, k_3_ = 4, here i = 1, γ_1_ = 1 & γ_2_ = 1.
  • k_1_ = 9, k_2_ = 6, k_3_ = 5, here i = 1, γ_1_ = 1 & γ_2_ = 1.
  • k_1_ = 10, k_2_ = 7, k_3_ = 3, here i = 1, γ_1_ = 1 & γ_2_ = 1.
  • k_1_ = 10, k_2_ = 6, k_3_ = 4, here i = 1, γ_1_ = 1 & γ_2_ = 1.
    S_1_ = [1,2,3,4,5,18,17,16,15], S_2_ = [6,7,8,13,12], S_3_ = [9,10]t are the sets of shifts to obtain MCBND for v = 20, k_1_ = 10, k_2_ = 6, k_3_ = 4.

## 4 Summary of the work with direction for future research

The constructors that are currently available in the literature to generate MCBNDs and their useful classes do not have the constructor development procedure. Assuming equal left- and right-neighbor effects, the process for developing the constructors that provide the full solution of MCBNDs, MCSBNDs, MCPBNDs-I, MCSPBNDs-I, MCWBNDs-I, and MCSBGNDs-I has been thoroughly explained in this article. These constructions are for:

• *v* *=* 2*i*k + C_1_ (equal block size)
• *v* = 2*i*k_1_+ 2γ_1_k_2_+ … + 2γ_h-1_ k_h_ + C_1_ (unequal block sizes), where *i* sets of shifts will be for k_1_ and γ_*j*_ sets for k_*j*_*.*

The process for developing the constructors outlined here will serve as the foundation for developing new constructors for designs with additional significant features. When left-neighbor effects and right-neighbor effects are not equal, constructors are developed which provide the full solution for:

(a) MCBNDs and MCSBNDs on the basis of Rule I for:
  • *v* (odd) = *i*k + C_1_ (equal block size)
  • *v* (odd) = *i*k_1_+ γ_1_k_2_+ … + γ_h-1_ k_h_ + C_1_ (unequal block sizes), where *i* sets of shifts will be for k_1_ and γ_*j*_ sets for k_*j*_, where C_1_ = 1 for MCBNDs and C_1_ = −1 for MCSBNDs.
(b) MCBNDs and MCNSBNDs on the basis of Rule II for:
  • *v* (integer) = *i*k + D_1_ (equal block size)
  • *v* (integer) = *i*k_1_+ γ_1_k_2_+ … + γ_h_k_h_ + D_1_ (unequal block sizes), where *i* sets of shifts will be for k_1_ and γ_*j*_ sets for k_*j*_, where D_1_ = 0 for MCBNDs and D_1_ = −1 for MCNSBNDs.
(c) MCPBNDs-I, MCSPBNDs-I, MCWBNDs-I and MCSBGNDs-I on the basis of Rule I for *v* even with:
  • *v* = *i*k + E_1_ (equal block size)
  • *v* = *i*k_1_+ γ_1_k_2_+ … + γ_h-1_ k_h_ + E_1_ (unequal block sizes), where *i* sets of shifts will be for k_1_ and γ_*j*_ sets for k_*j*_, where E_1_ = 2, 1, 0 & −1 respectively for MCPBNDs-I MCSPBNDs-I, MCWBNDs-I and MCSBGNDs-I.

## 5 Proposed future directions for research

To obtain the generalized classes of MCBNDs for the cases that are not available in the literature, some new constructors might be created. An R-package could be created based on all of these constructors to produce these classes for specified values of v and k. In order to obtain MCBNDs and their generalized classes, some new constructors that also satisfy the conditions of treatment balance may be developed.

## CRediT authorship contribution statement

**Khadija Noreen:** Data curation, Conceptualization. **Javid Shabbir:** Methodology, Investigation. **M.H. Tahir:** Writing – original draft, Visualization. **Akbar Fardos:** Software, Resources. **Rashid Ahmed:** Writing – original draft, Data curation, Conceptualization. **Olayan Albalwi:** Writing – review & editing, Funding acquisition.

## Data availability

All data is present in the manuscript.

## Declaration of competing interest

The authors declare the following financial interests/personal relationships which may be considered as potential competing interests:Reports a relationship with that includes: Has patent pending to. If there are other authors, they declare that they have no known competing financial interests or personal relationships that could have appeared to influence the work reported in this paper.
